# Pharmacopeia Collegii Regii Medicorum Edinburgensis

**Published:** 1784

**Authors:** 


III. *Pharmacopeia Collegii Regii Medicorum Edinburgenſis.* 8vo. Edin. 1783. p. 269.

IN this new edition of the Edinburgh Diſpenſatory, which is the ſeventh, ſeveral of the articles inſerted in the edition of 1774 are omitted or altered, and other new ones added. The College have likewiſe changed the names of ſome of the compound medicines formerly adopted, for others which they think are more expreſſive of their nature; and, in directing the proportions of different ingredients, they every where exclude liquid meaſure, as being leſs accurate than meaſure by weight.

In the firſt part of the work, or *Materia Medica,* the following new articles are inſerted; viz. *acetum vini*; *aqua ſtillatitia florum aurantiorum Hiſpalenſium*; *arnica*; *balſamum Gileadenſe*; *cardamine*; *carica*; *caryophylla aromatica*; *caſſia*

*ſia lignea* ; *cinara hortenſis* ; *curſuta* ; *digitalis purpurea* ; *dolichos* ; *elaterium* ; *filix mas* ; *geoffræa* (cabbage bark); *ginſeng* ; *gratiola* ; *lichen iſlandicus* ; *lobelia* ; *oleum ſtillatitium florum aurantiorum Hiſpalenſium* ; *olea expreſſa ſeminum lini*, *nucis moſchatæ* (*oleum macis* vulgo *dicti*), & *olivarum* ; *oliva* ; *palma* ; *pix liquida* ; *quaſſia* ; *radix indica lopeziana* ; *rhododendron* ; *ſal alkalinus fixus foſſilis* ; *ſalix* ; *ſanguis draconis* ; *ſapo albus Hiſpanus* ; *ſpigelia* ; *vipera* ; *ulmus.*

In the former edition many of the articles were either not defined at all or imperfectly deſcribed. This defect is now ſupplied with great accuracy with regard to the following ; viz. *Aloe hepatica* ; *aloe Socotorina* ; *amygdalæ dulces* ; *aſſafœtida* ; *aurantium Hiſpalenſe* ; *balſamum Canadenſe* ; *balſ. Copaibæ* ; *balſ. Peruvianum* ; *balſ. Tolutanum* ; *benzoinum* ; *camphora* ; *canella alba* ; *cantharides* ; *cardamomum minus* ; *caſcarilla* ; *caſſia fiſtularis* ; *caſtoreum* ; *cicuta* ; *cinnamomum* ; *cochinnilla* ; *colocynthis* ; *contrayerva* ; *cortex Peruvianus* ; *curcuma* ; *fœnum Græcum* ; *galbanum* ; *gallæ* ; *gambogia* ; *gummi Arabicum* ; *gummi Tragacantha* ; *jalapa* ; *ipecacuanha* ; *iris Florentinus* ; *manna* ; *maſtiche* ; *millepedæ* ; *moſchus* ; *oculi cancrorum* ; *olibanum* ;

*opium* ;

*opium*; *pimenta*; *piper longum*; *piper nigrum*; *pix Burgundica*; *pyrethrum*; *ricinus*; *rheum*; *ſantalum citrinum*; *ſantalum rubrum*; *ſantonicum*; *ſarſaparilla*; *ſaſſafras*; *ſcammonium*; *ſeneka*; *ſenna*; *ſerpentaria Virginiana*; *ſimarouba*; *ſpermaceti*; *ſtyrax calamita*; *tamarindus*; *terebinthina Veneta*; *terra Japonica*; *zedoaria*; *zingiber*.

The articles omitted are *ambragriſea*; *aurantium Curaſlavenſe*; *cornu cervi calcinatum*; *creta alba*; *crocus*; *eryngium*; *hypericum*; *lichen cinereus terreſtris*; *nux moſchata condita*; *opoponax*; *oſtreorum teſtæ*, *papaver album*; *ſambucus*; *ſymphytum*.

In the ſecond part of the work, which includes the *Medicamenta Preparata & Compoſita*, the additions and alterations are likewiſe conſiderable. The additional preparations are *aqua calcis*; *vinum e tartaro antimonali* (made by diſſolving 24 grains of emetic tartar in a pound of white wine); *aqua deſtillata*;—*aquæ ſtillatitiæ corticis malorum limoniorum & aurantiorum Hiſpalenſium*; *oleum e cornubus rectificatum*, ſive *oleum animale*;—*ſal alcalinus fixus foſſilis purificatus*;—*ſoda tartariſata*, vulg. *ſal rupellenſis*;—*vitriolum album*;—*ſolutio mercurii ſublimati corroſivi* (ſix grains of *merc. corroſ. ſublim.* and twelve of

*laſ*

*ſal ammoniac.* diſſolved in a pound of diſtilled water); —*pulvis mercurii cinereus* *; —*pilulæ ſcilliticæ* †; — *pilulæ ſtomachicæ*; — *linimentum ſimplex* (compoſed of four parts of oil of olives, and one of white wax); *unguentum ſimplex* (with five parts of oil and two of wax); *ceratum ſimplex* (with ſix parts of oil, and three of wax); —*unguentum e calce zinci* (compoſed of ſix parts of *linim. ſimpl.* and one of calx of zinc); —*un-guentum*

* R. Hydrargyri,

Acidi nitroſi tenuis, paria pondera.

Miſce ut ſolvatur hydrargyrus, ſolutum aqua pura dilue, & adde ſpiritus ſalis ammoniaci quantum ſatis ſit ad hydrargyrum penitus ab acido liberandum; pulvis dein aqua pura lavetur & exſiccetur.

† R. Gummi ammoniaci,

Cardamomi minoris in pulverem tenuem triti,

Extracti glycyrrhizæ, ſingulorum drachmam unam,

Radicis ſcillæ exſiccatæ, & in pulverem tenuem redactæ, ſcrupulum unum.

M. & ſyrupo ſimplice fiat 1

*guentum citrinum* ‡; — *unguentum e ſulphure* ſive *antipſoricum.*

The preparation of ſeveral of the ointments is rendered more ſimple in this than in the former edition. The *ceratum e lapide calaminare* is now directed to be made by mixing one part of the prepared *lapis calamin.* with five of the *ceratum ſimpl.* In the ſame manner the *unguentum e tutia* is to be made by incorporating one part of prepared tutty with five of *liniment. ſimpl.* and the *ung. ex ærugine* by mixing one part of verdigris with fifteen of *ung. baſilic.* The *ung. e pice*, inſtead of being compoſed of equal weights of liquid pitch and mutton ſuet, as in the former edition, is now directed to be prepared of five parts of the former and two of yellow wax.

The preparations omitted in this new edition are the following; viz. *Succi ſpiſſati flammulæ*

‡ R. Hydrargyri, unciam unam.

Spiritus nitri, uncias duas.

Digere ſuper arenam calidam ut fiat ſolutio, quæ calidiſſima adhuc miſceatur cum axungiæ porcinæ liquefactæ, & denuo frigeſcentis, libra una; ſtrenue dein miſturam ſubige in mortario vitreo, ut fiat unguentum uniforme.

*Jovis*

*Jovis & hyoſcyami ;—infuſum foliorum flammulæ Jovis ;—Vinum dictamni albi ;—tinctura meconii ;—aquæ ſtillatitiæ caſſiæ ligneæ & pulſatillæ nigricantis ;—aqua raphani compoſita ;—vitriolum album purificatum ;—cinnabaris factitia ;—pulvis antilyſſus ;—unguentum cereum* ſive *album ;—unguentum emolliens ;—emplaſtrum antihyſtericum.*

The articles of which the names are changed are comprized in the following tables.

| OLD NAMES. | NEW NAMES. |
|---|---|
| *Sal lixivius purificatus.* | *Sal alcalinus fixus vegetabilis purificatus.* |
| *Tartarum vitriolatum.* | *Alcali fixum vegetabile vitriolatum.* |
| *Sal catharticus Glauberi.* | *Soda vitriolata.* |
| *Tartarum ſolubile.* | *Alcali fixum vegetabile tartariſatum.* |
| *Tartarum regeneratum.* | *Alcali fixum veg. acetatum.* |
| *Cauſticum lunare.* | *Sal argenti.* |
| *Saccharum Saturni.* | *Sal plumbi.* |
| *Tartarus emeticus.* | *Tartarus antimonialis.* |
| *Syrupus e ſucco limonum.* | *Syrupus e ſucco malorum limoniorum.* |
| *Pilulæ cæruleæ.* | *Pilulæ e cupro.* |

| OLD NAMES. | NEW NAMES. |
| --- | --- |
| *Pilulæ mercuriales.* | *Pilulæ e hydrargyro.* |
| *Unguentum mercuriale.* | *Unguentum ex hydrargyro* five *cæruleum.* |
| *Unguentum epifpafticum mitius.* | *Unguentum epifpafticum ex infufo cantharidum.* |

